# Ferdinand Karl Piëch: A Psychobiography of a Ruthless Manager and Ingenious Engineer

**DOI:** 10.3389/fpsyg.2021.671243

**Published:** 2021-09-21

**Authors:** Claude-Hélène Mayer, Roelf van Niekerk, Nicola Wannenburg

**Affiliations:** ^1^Department of Industrial Psychology and People Management, University of Johannesburg, Johannesburg, South Africa; ^2^Kulturwissenschaftliche Fakultät, Europa Universität Viadrina Frankfurt (Oder), Brandenburg, Germany; ^3^Department of Industrial and Organisational Psychology, Nelson Mandela University, Port Elizabeth, South Africa; ^4^Department of Psychology, Rhodes University, Makhanda, South Africa

**Keywords:** psychobiography, dark sides of personality, Theodore Millon, personality prototypes, Millon personality spectrometer

## Abstract

The dark sides of personalities have gained importance during the past two decades. Psychobiography deals with the life of extraordinary individuals throughout the life span by applying selected theories to analyse specific life aspects. This study uses Theodore Millon’s (1990, 2011) personality theory to explore the life of Ferdinand Karl Piëch (1937–2019), an Austrian engineer and business executive who was the chairman of the executive board of the Volkswagen Group (1993–2002) and the chairman of the supervisory board from 2002 to 2015. Piëch was also known for having a complex and controversial personality. This study aims to explore the life and work of Ferdinand Karl Piëch through the lens of Millon’s personality theory. This study has four specific aims. Firstly, to provide an accurate and objective description of Piëch’s life history. Secondly, to formulate an accurate description of Piëch’s personality on the basis of existing biographical data. Thirdly, to interpret Piëch’s personality through the use of Millon’s theoretical framework and identify the personality prototypes that correspond with his personality characteristics. Fourthly, to contribute to expanding the field of “dark personality aspects” from a psychobiographical perspective, especially the personalities of extraordinary individuals. The findings suggest that although Piëch demonstrated the characteristics of three personality prototypes, the assertive-sadistic prototype is the best fit for his personality characteristics. Conclusions are drawn and recommendations for theory and practice are given.

## Introduction

The so-called dark side of personalities have gained interest in interdisciplinary research during the past decades (Paulhus and Williams, 2002; Kaufman, 2019; Czarna and Zajas, 2020). The idea of the dark personality was initially proposed by Paulhus and Williams (2002). They highlighted the dark triad of personalities, consisting of narcissism (entitled self-importance), *Machiavellianism* (strategic exploitation and deceit) and *psychopathy* (callousness and cynicism). Since then, several studies have linked negative personality traits to both positive and negative psychosocial outcomes (e.g., Kaiser et al., 2014). As a result, the focus of the dark sides of the personality are no longer merely directed at criminals and psychopaths as this dark triad can, to a certain degree, be found in each and every individual (Paulhus and Williams, 2002).

During the past two decades research has explored different aspects of dark personality traits (Paulhus and Williams, 2002; Kaufman, 2019; Czarna and Zajas, 2020). Most of this research contributes to a more in-depth exploration of the dark patterns of thoughts, emotions and behaviours. The research also corresponds with other research which looks primarily at the dark side of human nature in general. Kaufman (2019) points out that the negative sides of the dark pyramid is negatively correlated with, for example, compassion, self-transcendent values, agreeableness, conscientiousness, empathy, and the belief that humans and the self are good. In contrast, the dark sides of the personality have been positively correlated with utilitarian moral judgement, strengths of creativity, bravery and leadership, power, motivation to gain power, self-enhancement, and achievement (Kaufmann and Kaufmann, 2014; Holm-Hadulla et al., 2020).

Recent literature explores dark personality aspects in organisations, (Schyns et al., 2019; Czarna and Zajas, 2020; Ray et al., 2020), career success (Oflu et al., 2020) and leadership (Van Scotter, 2019; Mackey et al., 2020; Syed et al., 2020). Spurk et al. (2016) observed that narcissism as a dark personality trait is positively related to salary, Machiavellianism is positively related to leadership position, and career satisfaction while psychopathy is negatively related to career outcomes. Harms and Spain (2015, p. 15) indicate that there is still a huge void in understanding the “breadth and depth of the impact on dark personality in the workplace.”

This study aims to contribute to research on the dark sides of personality from a psychobiographical perspective. The focus will not be on criminal or deviant individuals as in previous studies (Paulhus and Williams, 2002), but rather on an exceptionally successful business person, namely Ferdinand Karl Piëch (1937–2019). Piëch was an Austrian engineer and business executive who was the chairman of the executive board of the Volkswagen Group (1993–2002) and the chairman of the supervisory board (2002–2015). Apart from being known for his successful business career in the automotive industry, Piëch was also known for his ruthless behaviour and management style (McGee and Milne, 2019).

## Psychobiographical Research

This study is based on a psychobiographical, case study approach. According to Mayer and Kőváry (2019, p. 1) “[p]sychobiography is the systematic application of scientific psychology in the interpretation of life and works of significant people like artists, scientists, philosophers, activists or politicians.” Psychobiographical researchers explore the lives and contributions of extraordinary individuals (Schultz, 2005). They believe that a holistic approach to unique, significant individuals is valuable and that most individuals are worth knowing in a profound way (Ponterotto, 2014).

The influence that psychobiographies exert within the field of psychology has become more extensive (Wegner, 2020). They often focus on successful extraordinary individuals who have contributed positively to the lives of others and society through writing, science, acting, politics, social care or entrepreneurship (e.g., Kováry, 2011; Mayer, 2017; Mayer and van Niekerk, 2020). However, in recent years, psychobiographies have also considered extraordinary individuals with dark personality traits as the subjects of their research, e.g., the mass murderers/serial killers Jeffrey Dahmer (Chezé, 2009) and John Gacy (Pieterse, 2012), and politicians, such as Donald Trump (McAdams, 2020). Psychobiographies usually explore, re-frame and re-narrate the life of a certain individual from a specific theoretical perspective (Fouché and van Niekerk, 2010). For many years, the theoretical approach was mainly influenced by psychoanalytical theories which led to psychobiographies being criticised for being influenced by theories that focus excessively on personality aspects which are deemed negative or ascribed to negative social outcomes (Mayer and Leichtman, 2012; Mayer and Kőváry, 2019).

This study uses Theodore Millon’s (1990, 2011) evolutionary personality theory to explore the personality and contributions of Piëch. Millon’s theory has been chosen as it provides a system of analysis which meaningfully interweaves the disparate but systemic parts of personality, such as the cognitive, the biophysical and interpersonal aspects. Millon (2011) suggests that motivating aims are active in each and every personality. These aims are arranged along three bipolarities: existence (i.e., the pleasure-pain polarity), adaptation (i.e., the active-passive polarity) and replication (i.e., the self-other polarity). He further defined and devised a set of personality patterns (Millon, 1990, 2011) which have since been well researched. Recent studies have also investigated Millon’s personality patterns in the context of leadership (e.g., Lilienfeld et al., 2019).

## Millon’s Evolutionary Personality Theory

Theodore Millon (1928–2014) was a leading personality theorist known for his many contributions to several disciplines, such as anthropology, art history, philosophy, and psychology. Millon was a visionary, “grand personality theorist” (Pincus and Krueger, 2015, p. 2). He focussed on the formulation of comprehensive theories of personality and psychopathology as well as the integration of different theoretical orientations. He attracted international attention when he was invited as the psychological representative on the American Psychiatric Association Task Force that revised the Diagnostic and Statistical Manual of Mental Disorders in the early 1980s.

During his career Millon authored more than 30 books characterised by pantheoretical, integrative, interdisciplinary reviews formulated in “accessible, rich, and clinically evocative psychological portraitures” of personality patterns (Pincus and Krueger, 2015, p. 2). Much of his career focussed on the development of a model of personality which was described successively as a biosocial, biopsychosocial, and finally as an evolutionary perspective (Pincus and Krueger, 2015).

### Key Constructs

Millon’s theory of personality comprises several key constructs including evolutionary drives, attribute domains and personality prototypes. These will be discussed further.

#### Evolutionary Drives

Millon’s model of personality describes the structure and styles of personality with regards to evolutionary drives (Grossman, 2015). He believed that personality is influenced by the interaction between individuals’ genetic make-up, their actual environment, other individuals, and their social experiences (Millon, 2011). These variables interact and personality develops as a result of an individual’s adaptation or preferred way of coping with the world. Individuals employ strategies to cope with four important evolutionary drives of which each represents a conflict or a polarity that require resolution. The drives are existence, adaptation, replication, and abstraction. Ultimately, the strategies that individuals use within each evolutionary drive, along with the deficiencies, imbalances and conflicts that emerge while coping with that drive, reflect the individual’s personality pattern (Grossman, 2015).

The first drive, *existence*, is associated with the pleasure-pain polarity. Essentially, the drive is associated with the question: What reinforcement does the individual seek (i.e., pleasure enhancement or pain avoidance)? Individuals who are primarily orientated toward pleasure enhancement tend to engage with experiences that improve quality of life, cause satisfaction, and pursue positive reinforcement. Those who are primarily orientated toward pain avoidance engage with experiences that protect them from threatening environments as well as negative sensations and emotions.

The second drive, *adaptation*, is associated with the passive-active polarity. This drive is associated with the question: How does the individual gain reinforcement (i.e., passively or actively)? Those individuals who cope with this drive through adjusting passively to the environment, do not use initiative to modify events. Instead, they prefer to wait for circumstances within their environment to happen before taking any initiative. They display characteristics such as being inactive, compliant, and restrained. Individuals who cope through active adaptation demonstrate a tendency to modify their surroundings (Grossman, 2015) and are characterised by vigilance, energy, and drive.

The third drive, *replication*, is associated with the self-other polarity and focuses on reproductivity and ultimately ensuring survival of the species. This drive is associated with the question: Who does the individual turn to for reinforcement (i.e., self or others)? Individuals who cope with strategies primarily concerned with the self, use egocentric tendencies associated with multiple partners and offspring. These individuals tend to be insensitive, inconsiderate, and uncaring and cope by focussing on others, value their affection and nurturance. On the other hand, individuals who cope with strategies primarily concerned with others is disposed toward nurturant-oriented actions that are seen as affiliative and protective. Consequently, they limit the number of partners and provide more affection to them. Grossman (2015) described these individuals as being empathic and communicative.

The fourth drive, *abstraction*, is not associated with a polarity, but rather with “sources employed to gather knowledge about life and the manner in which this information is registered and transformed” (Strack, 2005, p. 533). Abstraction refers to the capacity to symbolise or represent the environment (Grossman, 2015) and comprises competencies associated with higher order mental processes, rational decision making, executive functioning, and the ability to reflect.

In conclusion, Millon argued that personality represents a compromise of processes and systems that work together on the basis of the above-mentioned evolutionary principles or drives and that this compromise ensures the survival and actualisation of individual potential (Pincus and Krueger, 2015). This implies that deficiencies, imbalances, and conflicts can develop because of the specific strategies employed by individuals to meet the four evolutionary drives. This is an important factor to consider when assessing personalities.

#### Attribute Domains

Millon’s model (2011) encompasses eight attribute domains: expressive emotion, interpersonal conduct, cognitive style, intrapsychic dynamics, self-image, intrapsychic content, intrapsychic architecture, and mood/temperament. The attribute domains comprise four categories. Namely, behavioural aspects (i.e., expressive emotion and interpersonal conduct), phenomenological aspects (i.e., cognitive style and self-image), intrapsychic aspects (i.e., intrapsychic dynamics, intrapsychic content, and intrapsychic architecture), and biophysical aspects (i.e., mood/temperament). Millon also differentiated two types of attribute domains. Namely, functional and structural domains. These domains represent the criteria sets of the personality prototypes and allow for comparisons between different prototypes (Grossman, 2015).

Individuals differ with respect to the domains they enact most frequently. They differ not only in the degree to which they approximate each personality prototype, but also in the extent to which domains dominate their behaviour. Different domains will thus be dominant in different individuals, even when they share the same prototype. The functional and structural domains are subsequently described.

#### Functional Domains

The functional domains are expressive, observable, coping processes designed to regulate inner and outer life (Grossman, 2015). They include expressive emotion (i.e., overt behaviour arising from an affective state), interpersonal conduct (i.e., interactive style and relationships), cognitive style (i.e., method of organising and synthesising information from the environment), and intrapsychic dynamics (i.e., internal processes analogous to defence mechanisms and indirectly observable as acts of conflict resolution, needs gratification, and self-protection).

#### Structural Domains

The structural domains are deeper templates embedded within the personality and providing a base or platform for the functional domains (Millon, 2011). They are not as observable as the functional domains and include self-image (i.e., sense of self-as-object), intrapsychic content (i.e., general expectations of others as imprinted from early experience), intrapsychic architecture (i.e., organising structures of the psyche that gives insight to the strength and cohesion of a personality), and mood/temperament (i.e., neuropsychological functioning, general energy and affect characteristics, as well as physical health effects on mental functioning).

#### Personality Prototypes

Millon (2011) identified 15 personality prototypes (see Table 1 – Vertical columns). The prototypes emerge as individuals employ different strategies to cope with the polarities of evolutionary drives (Millon et al., 2010). In the process, core personality dimensions become apparent. They manifest in the form of imbalances due to overinvestment in one or more of the basic polarities, deficiencies, or conflicts and contain functional and structural domains. Essentially, these functional and structural domains form part of a prototype’s criteria set that guides assessment and intervention (Grossman, 2015; Millon et al., 2010). Because the same eight domains are present for each prototype (see Table 2), the prototypes are comparable by domain. The prototypes include both normal and pathological personality patterns (Grossman, 2015). Millon added a spectrum component to his model to provide an indication of the relative adaptiveness or maladaptiveness of a given personality prototype (see Table 1). Millon specified three levels of personality functioning – normal personality style, abnormal personality types, and clinical personality disorders. At the most adaptive end of each spectra, Millon describes normal personality styles. This level of functioning describes individuals who have a prototypal pattern that cope reasonably well and demonstrate flexibility in their primary motivating aims. Grossman (2015) suggested that normal functioning requires the ability to engage flexibly with strategies from both sides of the polarity depending on what is contextually appropriate. At the middle level of functioning, Millon described abnormal personality types that are less flexible and adaptive. Individuals who present with these personality types are likely to experience impairment in interpersonal interaction, self-reflection, temperament, and self-control that may create distress for themselves and others. At the most maladaptive level of functioning Millon described clinical personality disorders that describe individuals who have specific areas of personality vulnerability, maladaptivity, inflexibility, and interpersonal difficulties. These difficulties impose severe limits on the daily lives of individuals, such as relationship problems and psychological distress.

**TABLE 1:** Personality prototypes and respective spectra levels.

Normal style	Abnormal type	Clinical disorder
Adaptive	←——————————————————–→	Maladaptive
Apathetic	Asocial	Schizoid
Shy	Reticent	Avoidant
Dejected	Forlorn	Melancholic
Deferential	Attached	Dependent
Sociable	Pleasuring	Histrionic
Ebullient	Exuberant	Turbulent
Confident	Egotistical	Narcissistic
Aggrandising	Devious	Antisocial
Assertive	Denigrating	Sadistic
Reliable	Constricted	Compulsive
Discontented	Resentful	Negativistic
Abused	Aggrieved	Masochistic
Eccentric	Schizotypal	Schizophrenic
Unstable	Borderline	Cyclophrenic
Mistrustful	Paranoid	Paraphrenic

*Adapted from Grossman (2015).*

**TABLE 2:** Expressions of personality spectra across trait domains.

Trait domains	Expressive emotion conduct	Interpersonal	Cognitive style	Self-image	Content	Dynamics	Architecture	Mood/temperament
Schizoid	Impassive	Unengaged	Impoverished	Complacent	Meagre	Intellectualisation	Undifferentiated	Apathetic
Avoidant	Fretful	Aversive	Distracted	Alienated	Vexatious	Fantasy	Fragile	Anguished
Melancholic	Disconsolate	Defenseless	Fatalistic	Worthless	Forsaken	Asceticism	Depleted	Woeful
Dependent	Puerile	Submissive	Naive	Inept	Immature	Introjection	Inchoate	Pacific
Histrionic	Dramatic	Attention-seeking	Flight	Gregarious	Shallow	Dissociation	Disjointed	Fickle
Turbulent	Impetuous	High-spirited	Scattered	Exalted	Piecemeal	Magnification	Unsteady	Mercurial
Narcissistic	Haughty	Exploitive	Expansive	Admirable	Contrived	Rationalisation	Spurious	Insouciant
Antisocial	Impulsive	Irresponsible	Non-conforming	Autonomous	Debased	Acting out	Unruly	Callous
Sadistic	Precipitate	Abrasive	Dogmatic	Combative	Pernicious	Isolation	Eruptive	Hostile
Compulsive	Disciplined	Courteous	Constricted	Reliable	Concealed	Reaction formation	Compartmentalised	Solemn
Negativistic	Embittered	Contrary	Cynical	Discontented	Fluctuating	Displacement	Divergent	Irritable
Masochistic	Abstinent	Acquiescent	Diffident	Undeserving	Discredited	Exaggeration	Inverted	Dysphoric
Schizotypal	Peculiar	Secretive	Autistic	Estranged	Chaotic	Undoing	Fragmented	Distraught
Borderline	Spasmodic	Paradoxical	Vacillating	Uncertain	Incompatible	Regression	Split	Labile
Paranoid	Defensive	Provocative	Mistrustful	Inviolable	Unalterable	Projection	Inelastic	Irascible

*Adapted from Grossman (2015).*

### The Millon Personality Spectrometer

The Millon Personality Spectrometer (MPS) comprises eight tables – one for each domain – each containing 15 descriptive trait choices (Millon et al., 2010). The 15 descriptive traits for each domain were written to characterise individuals. Each trait is illustrated with characteristics and examples. MPS users are required to choose the best description of the individual in question (i.e., the 1st best fit). The individual being rated does not need to display precisely the characteristics that are listed. Instead, they need only be the best-fitting of the listed sets of traits. Because most people can be characterised by more than one set of traits, users have to also identify the second best fit as well as the third best fit (should there be another set of descriptive characteristics applicable to this individual). It is important to note that for rating normal individuals (i.e., those without a clinical disorder) who display only minor or mild aspects of a trait, users should nevertheless mark the best-fit columns and *not* leave any of the best-fit columns blank. Users must still fill them in, in rank order of best fit, even when the features of a trait are only marginally present. Users should continue to fill in their choices for all eight domains, one at a time, using the same procedure for each one. Table 3 summarises the spectra and their associated letters (A, B, C, etc.). Although the MPS requires that users further evaluate the individual they have just rated in terms of their current overall level of social and occupational functioning, this step had not been completed in this study. The reason is that the study is not a clinical or diagnostic study, but rather a study aiming at the accurate description of the personality characteristics of an extraordinary individual. The study offered the authors an opportunity to explore the usefulness of Millon’s theory and the MPS in psychobiographical research.

**TABLE 3:** Piëch’s spectrometer ratings.

	Attribute Ddmains							
	I	II	III	IV	V	VI	VII	VIII
Prototypes	Expressive emotion	Interpersonal conduct	Cognitive style	Self-image	Mood/Affect	Intrapsychic dynamics	Intrapsychic contents	Intrapsychic architecture
A. Apathetic–schizoid								
B. Schizotypal–schizophrenic								
C. Withdrawn–avoidant								
D. Attached–dependent								
E. Exuberant–turbulent	3			3				
F. Sociable–histrionic								
G. Confident–narcissistic	2	2		2		2		3
H. Paranoid–paraphrenic		3	2		3	3	3	
I. Non-conforming–antisocial			3		2		2	2
J. Assertive–sadistic	1	1	1	1	1	1	1	1
K. Dol1eful–melancholic								
L. Aggrieved–masochistic								
M. Resentful–negativistic								
N. Borderline–cyclophrenic								
O. Compliant–compulsive								

### Critique of Millon’s Theory

Although Millon’s theory is not above criticism, there is little doubt that he made a substantial contribution and left a lasting legacy. Grossman (2015) described Millon as one of the most influential figures in conceptualising personality in the latter 20th and early 21st centuries. Millon’s first contribution is the formulation of a comprehensive evolutionary model of personology that views individuals within their biopsychosocial contexts (Grossman, 2015). This model provided Millon with the basis or foundation for the publication of more than 30 books, the development of seven psychometric instruments, the founding of two journals (i.e., *Journal of Personality* and *Journal of Personality Disorders*), the co-founding of the International Society for the Study of Personality Disorders, as well as for his contributions to the development of earlier versions of the Diagnostic and Statistical Manual of Mental Disorders (Pincus and Krueger, 2015). The model also provides clinicians and researchers with a comprehensive framework to identify and describe normal and abnormal personality patterns.

However, the comprehensiveness of Millon’s theory also relates to its limitations. The theory made it difficult, if not impossible for him to pursue data-driven theorising and research. Basically, Millon was unable to collect data to test many of the aspects he wrote about. This resulted in him becoming overinvested in theoretical or deductive methods in an era when empirical procedures and inductive psychological science dominated research and training (Pincus and Krueger, 2015). Despite the limitations related to the model’s empirical structure, Millon was recognised for his lifetime achievements in clinical science and practice by multiple scientific societies, including the American Psychological Association, American Psychological Foundation, and the Society for Personality Assessment (Pincus and Krueger, 2015).

## Piëch’s Life and Career

Ferdinand Piëch (1937–2019) was the third of four children of Anton and Louise Piëch, a Nazi couple (Ewing, 2019) who lived in Austria. Anton ran the VW plant founded by Adolf Hitler during World War II while Louise was the daughter of Ferdinand Porsche, inventor of the Beetle and the luxury Porsche brand (Zeller, 2019). Piëch was a reluctant learner and performed poorly during his school years in Austria. His parents then sent him to a strict Swiss boarding school (1952–1958). Here, he continued to perform poorly due to dyslexia. Piëch experienced the boarding school as tough and described it as a “dark time” during which his personality hardened (Zeller, 2019), he was forced to be self-reliant, and decided that it is better not to rely on people (BBC, 2019). After school Piëch attended a Swiss university where he studied mechanical engineering. Here, he endured another strict system, but this time he performed much better. He graduated in 1962 with a master’s thesis on the development of a Formula One engine.

After graduating Piëch joined Porsche (1963–1971). Here he worked in the racing programme and later became technical manager. One of his first projects at Porsche was the design of the Porsche 917, one of the most successful cars ever built. Sales of the 917 increased dramatically because of its success in car races and especially after it won the legendary 24 Hours Le Mans. From the start of his career Piëch demonstrated perfectionism and an obsession with details. He was also prepared to invest energy and a lot of money in his projects. The latter created friction with family members also involved at Porsche (Viehöver, 2019). Piëch left Porsche after intense family conflicts which led to a policy decision that no Porsche family member would in future be allowed to be involved in the management of the company (Hetzner, 2017; Ewing, 2019).

Piëch was now unemployed. He soon established a small engineering company where he developed a five-cylinder diesel engine for Mercedes-Benz. Piëch then joined Audi in 1972. Audi was part of the Volkswagen group, but not controlled by Porsche-Piëch clan, so for the first time in his career Piëch was employed outside the family business. Within 3 years he was appointed Manager of Technological Engineering (Hetzner, 2017). In the early 1970s, Audi was perceived as a reliable, but unremarkable brand (BBC, 2019). Through technical innovation Piëch contributed to Audi’s transformation into a popular luxury brand that competed with two other luxury German brands, BMW and Mercedes-Benz (Ewing, 2019). For example, he introduced four-wheel passenger vehicles (the so-called Quattro brand), five-cylinder petrol engines, diesel engines for passenger vehicles (e.g., the popular TDI engines), as well as lightweight aluminium bodies with exceptional aerodynamic capabilities. These innovations helped Audi to compete aggressively with the existing market-leaders (Horrell, 2019). From then on, technical innovations, or “Vorsprung durch Technik” became Piëch’s trademark. During the 20 years, Piëch worked at Audi (1972–1992) and where he later became the Chairman (in 1988), he masterminded an almost complete branding transformation that positioned Audi as high-performance, luxury cars (BBC, 2019).

His success at Audi motivated the VW Supervisory Board to name Piëch as CEO and Chairman of the Executive Board of VW Group in 1993 (Ewing, 2019). He held this position until 2002 when he had to retire at age 65 years after which he became Chairperson of the Supervisory Board. When Piëch joined VW, the company suffered massive losses and experienced problems with poor quality, high costs, and limited market appeal. In the United States specifically, VW lost its market position. These problems brought VW to the brink of bankruptcy (Ewing, 2019).

As was the case at Audi, Piëch played a central role in orchestrating a spectacular turnaround. The turnaround involved a series of interventions: the dismissal of almost the entire management board, the streamlining of production, increasing sales by producing better vehicles and rapid international expansion, cutting costs by persuading union leaders to accept a shortened workweek as well as ingenious engineering innovation. For example, Piëch introduced the combination of engine computers and fuel injection to improve fuel efficiency. Similarly, under his leadership VW successfully reduced engine noise in diesel cars and improved the smell of diesel emissions. Piëch also masterminded the platform strategy (Horrell, 2019; Zeller, 2019), a modular system that allow multiple brands and models to share chassis, electronic, and body-structure systems (Kacher, 2019). This strategy streamlined research, development, purchasing, production and resulted in massive cost-saving (Ewing, 2019). It provided VW with an opportunity to invest more money into the development of better interiors and a wider model range. The platform strategy has since become standard practice among multi-brand car companies. The above-mentioned interventions (as well as the development of influential models, such as the New Beetle and the first one litre engine in the world) guided by Piëch helped VW to return to profitability without job cuts and improved his relationships with shareholders and unions, providing him with a cult-like following. This allowed him to get his way during conflicts with colleagues and when he unilaterally decided to dismiss executive managers.

The two decades Piëch led VW were indeed characterised by extraordinary success, but also by reputational damage to the brand (Ewing, 2019; Zeller, 2019). This contributed to the complicated legacy Piëch left (Kacher, 2019).

Piëch rescued Volkswagen from a financial crisis and turned it into a large conglomerate comprising no less than 13 automotive brands (such as Bentley, Bugatti, Ducatti, Lamborghini, MAN, Porsche, Rolls Royce, Scania, Seat, and Skoda) and the world’s biggest manufacturer of motor cars (Horrell, 2019; Winton, 2019; Zeller, 2019). This achievement was due to his engineering brilliance; personal involvement in vehicle development; strong and clear direction; unwavering commitment to excellence; an obsession with manufacturing excellent cars; competitiveness; a growth-strategy based on the expansion of model ranges, acquisitions, and internationalisation; and a visionary entrepreneurial outlook (Horrell, 2019).

However, the shadow-side of this achievement included several blunders and accusations. His biggest financial mistakes include the Audi 2, Bugatti Veyron (the fastest, most powerful, and most expensive road legal car ever built), and VW Phaeton (Winton, 2019), the three VW vehicles included in the list of 10 biggest financial failures in modern automotive history (Rauwald and Reiter, 2019). In addition, Piëch was accused of stealing corporate information from General Motors, making prostitutes available to VW union leaders to gain their support (Ewing, 2019), an attempt to appoint his spouse as supervisory board chairwoman to succeed him, and the so-called dieselgate scandal (Zeller, 2019). This corporate scandal represented the biggest crisis in VW’s history, cost VW a lot of money (more than €30-billion in penalties and settlements), damaged its image (Zeller, 2019), and contributed to a growing negative attitude toward diesel passenger cars. Dieselgate made the headlines in 2015 when it was discovered by controlling bodies in the United States that VW had fitted cars with software that falsely reduced exhaust emission data.

Piëch’s abrupt exit from the VW group in 2015 ended an extraordinary career of more than 50 years in the automotive industry. During this career, recognition from peers came in the form of three special awards: Car Executive of the Century (in 1999), Automobile Magazine Man of the Year Award (in 2011) and induction into the Automotive Hall of Fame (in 2014). He is also recognised as an engineer of extraordinary breadth (Horrell, 2019) who shaped the development of the entire automotive industry, and one of the most influential car executives of the last century (Hetzner, 2017; Ewing, 2019).

## Aim of the Study

The study aims at exploring the life and work of Ferdinand Karl Piëch, a successful Austrian engineer and business executive, through the lens of Millon’s personality theory. More specifically, this study has three aims. Firstly, to formulate an accurate description of Piëch’s personality on the basis of existing biographical data. Secondly, to interpret Piëch’s personality through the use of Millon’s theoretical framework and identify the personality prototypes that correspond with his personality characteristics. Thirdly, to contribute to expanding the field of “dark personality aspects” from a psychobiographical perspective, especially the personalities of extraordinary individuals.

## Research Methodology

### Research Design

The study is based on a psychobiographical single-case study design (Yin, 2018) formulated according to a modern hermeneutics research paradigm which deals with the philosophical theory of the interpretation of meaning (Dilthey, 2002). By employing psychological theories, models, or frameworks, psychobiographies explore the lives and contributions of individuals who appear to be extraordinary in terms of their impact on society, their work, creativity, or leadership (Fouché and van Niekerk, 2010; Mayer and Maree, 2017). Psychobiographical case studies represent a valuable research approach and method for in-depth exploration of the lives and contributions of extraordinary individuals (Schultz, 2005; Ponterotto, 2014) who then might act as role-models for individuals and societies. Psychobiography is now recognised as an appropriate research method for elucidating life data (Schultz, 2005; Mayer and Kőváry, 2019) and can contribute to the understanding of extraordinary leadership (Long, 2014).

### Psychobiographical Subject

Ferdinand Piëch was purposefully sampled (Musarrat Shaheen and Pradhan, 2019). He was chosen as a research subject based on the following criteria:

1. Piëch may be classified as an individual who meets the general characteristics of a subject for psychobiographical research (McAdams, 1988; Durrheim and Painter, 2006) as he made extraordinary contributions to the motor industry.
2. Piëch is generally known as an eccentric, ruthless, and extravagant individual (McGee and Milne, 2019) and this paper aimed at exploring the complexity of his personality traits more thoroughly through the use of Millon’s personality theory.
3. Piëch’s personality evokes interest based on the descriptions of his behaviour and the authors aimed to explore him as a business person, but also as an entrepreneur and a highly successful individual with special focus on integration of the light and dark sides of his personality.

### Data Collection, Analysis, Interpretation, and Reporting

The data collection included two data sources, namely primary (i.e., autobiographical) and secondary (i.e., biographical) data (Allport, 1961). The data on Piëch were collected, content-analysed and interpreted through the lens of Millon’s personality theory. The data were employed in this study to reconstruct the life of Piëch in the light of his fascinating personality traits and their positive and negative psychosocial outcomes. The authors used a process of content analysis, more specifically, the five-step-process of data analysis described by Terre Blanche et al. (2006), namely familiarisation and immersion, inducing themes, coding, elaboration, as well as interpretation and checking. The findings are presented in a qualitative reporting style.

### Quality (Trustworthiness) Criteria and Ethical Considerations

The study adheres to qualitative research criteria for psychobiographical studies (Schultz, 2005). In order for the study to be credible or trustworthy, the authors paid particular attention to validity and reliability concerns within a qualitative framework (Yin, 2018). While terms such as validity and reliability do not hold the exact same meaning for qualitative research as they do in the quantitative sense, the construct validity, rigour, and dependability of the study are all imperative aspects of psychobiographical research. These considerations in relation to this paper are all supported by the authors setting out the aims of the research as well as the details of the method that was used in order to collect and analyse the data (Long and Johnson, 2000; Yin, 2018). As with construct validity, the operationalisation of the theory used (in this case Millon’s theory) increases the dependability and rigour of the study (Van der Riet and Durrheim, 2006; Yin, 2018). Researcher bias pertains to the credibility of a study and concerns addressing one’s own feelings about the subject as well as being careful not to make the subject comply with a particular theory. This was addressed by the authors being in continued reflective discussion with one another (regarding thoughts and feelings about Piëch) while collecting, analysing, and reporting on the data (Ngwenya and Manganyi, 2003; Yin, 2018).

The study follows well-established ethical considerations, such as using public resources exclusively (Runyan, 1988). The study further refrains from formulating a one-sided, potentially harmful or embarrassing portrayal of the research subject or his family (Runyan, 1988; Elms, 1994; Ponterotto, 2017a,b). The authors adopted a balanced and respectful attitude toward Piëch and took special care to ensure that his personality and contributions to the automotive industry were explored with empathy and responsibility (Ponterotto, 2017a,b).

## Findings and Discussion

This section comprises three sections. The first section provides a summary of descriptive statistics yielded by the MPS rating procedure. The second section focuses on the evolutionary drives and strategies of individuals who share Piëch’s personality characteristics and how they tend to cope with these drives. The third section focuses on the attribute domains and comprises four sub-sections. The first sub-section briefly describes the attribute domain, the second identifies the three prototypes relevant to Piëch’s domain characteristics, the third reviews the personality characteristics associated with the three prototypes, while the fourth discusses the biographical data that support the prototypical personality characteristics.

### Spectrometer Ratings

Table 2 indicates that although Piëch received ratings for five different prototypes, one particular prototype emerged as the one that approximates Piëch’s personality more closely than the other four. The ratings indicate that Piëch’s personality is best represented by the assertive-sadistic prototype. Piëch received eight ratings for the assertive-sadistic prototype, five ratings for the confident-narcissistic prototype, five ratings for the paranoid-paraphrenic prototype, four ratings for the non-conforming-antisocial prototype, and two ratings for the exuberant-turbulent prototype. These ratings confirm Millon’s (2011) contention that individual personalities represent a blend of two or more primary prototypes. In Piëch’s case, his personality is mainly represented by the assertive-sadistic, confident-narcissistic, and paranoid-paraphrenic prototypes.

### Evolutionary Drives

In this section, the first three drives (those drives that are associated with polarities) are discussed. The first drive, *existence*, is associated with the pleasure-pain polarity and asks the questions: What reinforcement does the individual seek (i.e., pleasure enhancement or pain avoidance)? Individuals who present with the characteristics of the assertive-sadistic prototype demonstrate a reversal of the properties associated with the pain-pleasure polarity. This prototype fits into the intrapsychically conflicted spectra category and represent those who cannot experience the life-enhancing qualities of the pleasure polarity. They prefer pain (e.g., stress, fear, and cruelty) rather than pleasure as their primary mode of relating to others. They engage in behaviours that are at odds with the typical aims of the pleasure polarity, that is, seeking joy, optimism, and pleasure in relating to one’s environment. In essence, these individuals remain at war with themselves. The conflict they experience with regards to the pain-pleasure polarities implies that they view pleasurable experiences as painful and painful experiences as pleasurable. Millon (2011) provided insight into the motivation for this behaviour. He indicated that individuals who demonstrate assertive-sadistic personality traits tend to anticipate hostility from others. Their characteristic aggressive behaviour is thus an attack with which they counter the meanness and humiliation they have learned to expect. In fact, they prefer to attack first and seize as much power as possible to prevent others from exploiting them.

The second drive, *adaptation*, is associated with the passive-active polarity and asks the questions: How does the individual gain reinforcement (i.e., passively or actively)? (Grossman, 2015). Individuals who present with the characteristics of the assertive-sadistic prototype cope through active adaptation by modifying their surroundings. They are characterised by their vigilance, energy, and drive. Furthermore, they act in a hostile and malevolent manner, actively working toward harmful and ruinous ends.

The third drive, *replication*, is associated with the self-other polarity and focuses on reproductivity and the ultimate survival of the species. This drive is associated with the question: Who does the individual turn to for reinforcement (i.e., self or others)? (Grossman, 2015). Individuals who present with the characteristics of the assertive-sadistic prototype are primarily concerned with the self and therefore typically employ egocentric strategies (Grossman, 2015). Millon claimed that these individuals tend to be insensitive, inconsiderate, and uncaring and consequently often degrade others and imposes pain. Rather than uplifting and preserving life, these individuals are actively evil and violent, assaulting, and demeaning others instead of encouraging and enhancing them.

### Attribute Domains

In the next section the eight domains will be presented and discussed. It is important to keep three principles relating to domain characteristics in mind. Firstly, individuals differ with regards to the domains they enact most frequently (Millon et al., 2010). Secondly, individuals differ in the degree to which they resemble a specific personality prototype. Thirdly, they differ in the extent to which each domain dominates their behaviour. Therefore, it is to be expected that different domains will be dominant in different individuals, even when those individuals share the characteristics of the same prototype (Millon et al., 2010).

#### Expressive Emotion Domain

This domain relates to the observable behavioural level of emotion. The characteristics of the *assertive-sadistic* prototype best fit Piëch’s behaviour. The biographical data suggests that he also demonstrates characteristics of two other prototypes. Namely, the *confident–narcissistic* and *exuberant–turbulent* prototypes. Millon et al. (2010) describe individuals who demonstrate the behavioural characteristics of the assertive-sadistic prototype as abrupt, reckless, callous, unflinching (undaunted), undeterred by pain, and attracted to challenge. Individuals who demonstrate the behavioural characteristics of the confident–narcissistic prototype typically appear to view themselves as above conventional rules of shared social living. They typically view such rules as naive and not applicable to them. They also reveal an egocentric indifference to the needs of others (e.g., acts arrogantly, self-assured, and confident). Individuals who present with the exuberant–turbulent prototype are energetic, driven, excitable, and overzealous. They are typically unrestrained, hot-headed, and worked up.

The above description is supported by the biographical data. The data suggests that Piëch was typically impatient with and indifferent to the views or feelings of colleagues and employees. He was keen to impose his preferences and get his way (Viehöver, 2019). He dealt promptly with anyone who dared to oppose him (Hetzner, 2017). Millon et al. (2010) refer to this characteristic as “an egocentric indifference to the needs of others” (p. 401). One of Piëch’s trademarks was the tendency to fire or demote senior managers who failed to meet his standards (Ewing, 2019) or dared to question him (Winton, 2019). Piëch never listened to or explored reasons why goals had not been achieved. Instead, he viewed employees as expendable, replaced them without ceremony, in the process clearly communicating the message that he demanded submissiveness from everyone. This suggests that Piëch viewed himself as being “above conventional rules of shared social living, viewing them as naive or inapplicable to self” (Millon et al., 2010, p. 401). This leadership style led Bob Lutz, who served as top executive in three automotive manufacturers (Ford Motor Company, Chrysler Corporation, and General Motors), to the conclusion that although he respected Piëch, he would never want to work for or with him in any capacity (cited in Nunez, 2015).

Throughout his career, Piëch enjoyed a challenge. For example, some of Piëch’s risky business decisions or strategies involving the development of unusual vehicles puzzled his colleagues. This did not deter him. In some cases, his plans paid off and proved beneficial for the company, at other times the risk failed (George, 2015). Another trademark was a recklessness that characterised his approach. He enjoyed the speed and power of the cars they built (Viehöver, 2019) and was driven toward improving their capabilities. In the process, he left no stone unturned to push technical boundaries, sometimes with fatal consequences for drivers whom he also viewed as expendable (Ewing, 2019).

#### Interpersonal Conduct Domain

This domain relates to interpersonal style and includes behaviours, attitudes, strategies employed to ensure satisfaction of interpersonal needs, and the style of coping with interpersonal tensions and conflicts. Although the characteristics of the *assertive-sadistic* prototype best fit Piëch’s behaviour, the data suggests that his characteristics also demonstrate some of the characteristics of two other prototypes: *confident–narcissistic* and *paranoid–paraphrenic*.

Millon et al. (2010) indicated that individuals who demonstrate the behavioural characteristics of the assertive-sadistic prototype typically experience satisfaction in competing with, dominating, and humiliating others. Their communication is often abusive and derisive; and they tend to engage in harsh, physically brutal behaviour (e.g., intimidation, coercion, and degradation). Individuals who demonstrate the characteristics of the confident-narcissistic prototype are typically entitled, self-centred, unempathetic, and inconsiderate. They expect special favours without assuming reciprocal responsibilities while they take others for granted and exploit them for egocentric purposes. Individuals who demonstrate the characteristics of the paranoid-paraphrenic prototype tend to be provocative, quarrelsome, and distrustful. They also tend to bear grudges and question the loyalties and motives of others.

This description is supported by the biographical data. The data suggests that the interpersonal conduct domain probably represents one of Piëch’s areas of vulnerability or weakness. The data suggests limited social skills, a rude and unfriendly interpersonal style, an exaggerated need to manipulate and control others, as well as a tendency to compete, rather than cooperate.

Piëch’s awkwardness in social situations attracted attention as he moved up the leadership ladder at VW in the late 1980s and early 1990s. At the time his seniors tried to keep him out of the public eye by limiting his work activities and scope to a technical role (Keller, 1993). Co-workers and journalists found it difficult to interview and interact with him. Some indicated that it was impossible to hold a conversation because of the long silences that characterised Piëch’s communication style (Hetzner, 2017). Piëch himself struggled to relate, especially with the emotional aspects of communication processes (Piech, 2002).

Apart from his awkwardness during conversations, Piech was also known for his rude and unfriendly attitude toward others. He was described as a ruthless, autocratic manager who attached no value to collegiality within the workplace (Hetzner, 2017; Schudel, 2019). Instead, he used fear and intimidation to drive performance (Hetzner, 2017). Lutz described Piëch’s management style as “a reign of terror and a culture where performance was driven by fear and intimidation” (cited in Nunez, 2015).

Throughout his career Piëch employed a remarkable range of strategies to manipulate and control others. Zeller (2019) described him as a Machiavellian master and a master of intrigue. The term Machiavellian is used to describe individuals that are primarily focussed on their own interests and therefore manipulate, deceive, and exploit others to achieve their objectives (Paulhus and Williams, 2002). The manipulative strategies Piëch used were very effective and sustained his career. He always pursued a clear strategy (Viehöver, 2019) that relied on complete domination (Ewing, 2019). With this, he ensured that he got his way. Among the strategies employed by Piëch were aggression and ruthlessness (Winton, 2019; Zeller, 2019), intimidation and threats of dismissal, demanding specific goals and standards (Piech, 2002), spreading of rumours through the media (Viehöver, 2019), stirring up of in-fighting (Ewing, 2019), the use of connections to undermine relatives, the enlisting of unionists (whom he actually loathed) (Viehöver, 2019), and claiming ownership of other peoples’ ideas (Hetzner, 2017).

Piëch did not believe in cooperation. In his autobiography he admitted that his desire for harmony was limited (Piech, 2002). Instead, he was a merciless competitor (Hetzner, 2017). Horrell (2019) indicated that competition was the fuel for Piëch’s fire. He was keenly aware of the profits made by other automotive companies and this motivated him to aggressively compete with them. However, Piëch took competitiveness one step further. He incited competition between different divisions at VW to drive the performance of different teams (Horrell, 2019).

The data suggests that although Piëch’s interpersonal style represents one of his areas of vulnerability or weakness from a psychological perspective, it enabled him to leverage his success and reputation as a manager. His style was characterised by decades-long grudges and hostility between himself and his relatives, intense conflicts with stakeholders, power struggles with colleagues, as well as a complete lack of empathy for subordinates against whom he often turned (Ewing, 2019; Horrell, 2019).

#### Cognitive Style Domain

This domain relates to individuals’ distinctive way of thinking and includes key cognitive functions such as style of focussing, allocating attention, processing information, organising thoughts, and making attributions. Although the characteristics of the *assertive-sadistic* prototype best fit Piëch’s behaviour, the data suggests that his characteristics also demonstrate some of the characteristics of two other prototypes, namely *paranoid–paraphrenic* and *non-conforming–antisocial*.

Millon et al. (2010) listed the cognitive characteristics of the assertive-sadistic prototype as being opinionated, obstinate in holding to preconceptions, and exhibiting social intolerance and prejudice (e.g., closed minded and bigoted). Individuals who demonstrate the characteristics of the paranoid-paraphrenic prototype are typically mistrustful and suspicious of the motives of others. They tend to interpret events as signifying deceit, malice, or betrayal. Individuals who demonstrate the characteristics of the non-conforming–antisocial prototype tend to hold deviant and socially unorthodox beliefs and morals. At the same time, they show contempt for traditional ideals, conventional rules, and social morals.

This description is supported by the biographical data. The data suggests that the cognitive style domain represents one of Piëch’s areas of strength and that although Piëch undoubtedly demonstrated characteristics such as being opinionated, dogmatic, and intolerant, several cognitive characteristics contributed to his spectacular track record at Porche, Audi, and Volkswagen. Colleagues and relatives who worked with Piëch at Porsche experienced his dogmatic attention to detail, meticulousness, and tenacity soon after he started his first job as a designer (Viehöver, 2019). However, within a decade these characteristics contributed to Piëch being dismissed from the family business. Although Piëch played an important role in numerous Porsche victories at Le Mans, he was also responsible for considerable financial losses and intense conflict with siblings and cousins who worked in the family business (Hetzner, 2017). The intense family conflict that started early in Piëch’s career lasted until Piëch died. Piëch did not trust them and he trusted few others. He held a grudge against his cousins from his childhood. According to Piëch they had the privilege of attending a permissive Steiner school, while he, as a misunderstood dyslexic and reluctant learner had been sent to a very strict boarding school and university (Viehöver, 2019). Piëch’s inability to trust people extended to religious or spiritual matters (Piech, 2002). Piëch was an atheist and showed contempt for traditional or conventional morals and rules. Apart from disapproving of religion, his family situation also demonstrates his dislike for social convention. At the time of his death Piëch had 12 children with four different mothers, including the wife of his cousin.

In addition to entertaining unorthodox social beliefs and morals, Piëch’s cognitive style was also intolerant. He was quick to identify incompetence among subordinates and had no patience with them (Ewing, 2019). He also believed that it was not important to listen to the views of colleagues and did not tolerate being challenged or questioned (Winton, 2019). He viewed this as deceit and betrayal. Similarly, colleagues who could not answer his probing questions quickly and intelligently seldom lasted long in their job (Horrell, 2019).

As stated above, although Piëch’s dogmatism, stubbornness, and intolerance probably kerbed his cognitive style, other cognitive characteristics played an important role in the innovations, inventions, and turnarounds he orchestrated during his distinguished career. One of the characteristics that facilitated his spectacular successes was his obsessiveness. He obsessed about goals, production processes, quality, standards, detail as well as the big picture (Piech, 2002; Horrell, 2019). He was also fanatical about innovations that would move technological boundaries in automotive development forward. According to Hetzner (2017) the slogan of Audi, *Vorsprung durch Technik* was the personification of Piëch who judged that technology could solve all automotive problems. Viehöver (2019) concurred and referred to Piëch as a perfectionist technocrat, while Zeller (2019) described him as a brilliant technician and an engineer who had the ability to build cars by himself.

#### Self-Image Domain

This domain relates to individuals’ perception of their own distinct identities. Although the characteristics of the *assertive-sadistic* prototype best fit Piëch’s behaviour, the data suggests that his characteristics also demonstrate some of the characteristics of two other prototypes, namely the *exuberant–turbulent* and *confident–narcissistic* prototypes. Millon et al. (2010) indicated that individuals who demonstrate the characteristics of the assertive-sadistic prototype typically value an identity that is tough, domineering, power-oriented, unsympathetic, and unsentimental. They are often proud of the stern image they portray and how they are feared by others. Individuals who demonstrate the characteristics of the exuberant–turbulent prototype perceive themselves as energetic and full of vitality. They view themselves as hardy, robust, and enterprising. Individuals who demonstrate the characteristics of the confident-narcissistic prototype typically act in a self-assured manner. They present themselves as confident and are keen to display their achievements publicly. Despite their personal sense of self-worth, they are usually seen by others as self-centred, inconsiderate, and arrogant.

The description above is supported by the biographical data. Viehöver (2019) confirmed Piëch’s domineering and power-oriented approach that led to people fearing and avoiding him. Kacher (2019) confirmed this by stating that there has never been a more powerful *Automobile Magazine Man of the Year*. Furthermore, Ewing (2019) indicated that Piëch completely dominated Volkswagen. Hetzner (2017) described Piëch as a despot who surrounded himself by yes men. Not surprisingly, Piech (2002) too described himself as an aggressive and demanding manager. He knew exactly which goals he wanted to achieve and how he wanted to go about attaining them (Piech, 2002). Employees who did not cooperate with his goal-directed projects were dealt with harshly. As far as self-assuredness is concerned, Lutz (cited in Winton, 2019) commented that Piëch’s self-confidence came across as arrogance and that he dismissed many colleagues who dared to question him. Piëch certainly left no stone unturned to instil fear in subordinates. He ruled with an iron fist, threatened and dismissed co-workers, set demanding and specific goals and standards for projects, and also employed aggressive management methods that, according to Winton (2019), most people would find unacceptable today.

#### Mood/Affect Domain

This domain relates to individuals’ predominant affect (i.e., emotions, moods, and feelings) and the intensity and frequency with which they express it. Affect permeates individuals’ ongoing relationships and experiences and may be revealed indirectly in activity level, speech quality, and physical appearance.

Although the characteristics of the *assertive-sadistic* prototype best fit Piëch’s mood and affect, the data suggests that his characteristics overlap with some of the characteristics of two other prototypes, namely the *paranoid–paraphrenic* and the *non-conforming–antisocial* prototypes. Millon et al. (2010) indicated that individuals who demonstrate the mood and affect characteristics of the assertive-sadistic prototype are typically hostile, argumentative, bad tempered, and willing to do harm, even persecute others to get their own way. Individuals who demonstrate the characteristics of the non-conforming-antisocial prototype are uncaring and show deficits in empathy, compassion, guilt or remorse. Generally, they tend to be indifferent to the welfare of others. Individuals who demonstrate the characteristics of the paranoid–paraphrenic prototype may appear to be unemotional and objective but are quick to take offence and react angrily.

This description is supported by the biographical data. In his autobiography Piech (2002) made it clear that he did not value harmonious relationships or the emotions that support them. In fact, he indicated that once he has identified an important goal, he pursues it without paying attention to anything else. Ewing (2019) referred specifically to the quality of Piëch’s feelings toward subordinates and indicated that he did not empathise with them and also had no patience with them. The available data does not refer to a single situation where Piëch demonstrated remorse. During his career he was accused of a number of questionable behaviours, such as stealing corporate information, making prostitutes available to union leaders to gain their support (Ewing, 2019), attempting to appoint his spouse as supervisory board chairwoman, and fitting cars with devices programmed to provide false exhaust emission data (Zeller, 2019). Piëch adamantly refused to react to these allegations, despite the financial and reputational damage they brought to himself, VW, and members of his extended family (Viehöver, 2019). In other situations, he reacted to criticism by dismissing the critics (Hetzner, 2017). Piëch was notoriously difficult to get along with. He spoke very little, displayed a no-non-sense approach (BBC, 2019), and inspired considerable fear (Winton, 2019). In addition, he over-reacted to mistakes made by employees (Kacher, 2019). He was clearly indifferent to the welfare of employees. Piëch treated people who dared to oppose or criticise him very harshly, irrespective of whether they were relatives, stakeholders, or hand-picked successors. He used his network, manipulated people, and fuelled rivalries to persecute opponents, eliminate them, and get his own way (Viehöver, 2019).

Piëch demonstrated argumentativeness, bad-temperedness, and hostility toward others. He had an abrasive personality that resulted in constant bickering with his siblings and cousins. This carried on throughout his career and also involved subordinates, colleagues, and stakeholders (Horrell, 2019). For example, the enmity between Piëch and his cousin, Wolfgang Porsche, lasted for decades and had not been resolved during his lifetime (Horrell, 2019). Ewing (2019) aptly described him as a corporate infighter.

#### Intrapsychic Dynamics Domain

This domain relates to the defence mechanisms (i.e., internal regulatory processes) of self-protection, need gratification, and conflict resolution that may sometimes be consciously recognised, but usually represent data derived primarily at the intrapsychic level.

Although the characteristics of the *assertive-sadistic* prototype best fit Piëch’s intrapsychic dynamics, the data suggests that he also demonstrated some of the characteristics of two other prototypes, namely the *confident–narcissistic* and *paranoid–paraphrenic* prototypes. Millon et al. (2010) indicated that individuals who demonstrate the characteristics of the assertive-sadistic prototype use isolation as a primary defence and are typically cold-blooded and unaware of the impact of their destructive acts. They view individuals on the receiving end of their aggressive and destructive acts impersonally, often as symbols of devalued groups devoid of feelings. Individuals who demonstrate the characteristics of the confident–narcissistic prototype use rationalisation as a primary defence. They are typically self-deceptive and casual in rationalising self-centred and socially inconsiderate behaviours. Individuals who demonstrate the characteristics of the paranoid–paraphrenic prototype use projection as a primary defence. They typically disown undesirable personality characteristics and attribute them to others. They also tend to ignore their own weaknesses while remaining hypercritical of the weaknesses of others.

The data generally supports the above description. In the following section examples will be reviewed that illustrate Piëch’s cold-blooded approach and denial of the impact of his behaviour. Firstly, Piëch used dismissal as one of the methods to control people and silent dissent (Hetzner, 2017; Ewing, 2019; Winton, 2019). He seemed to have done so in a cold-blooded fashion without considering the implications of losing a job or an executive position in the world’s largest and most successful automotive company. Secondly, during his involvement with the Porsche racing car project he insisted on pushing technical boundaries even though it sometimes had fatal consequences for drivers (Ewing, 2019). Thirdly, Piëch represents the third generation after Ferdinand Porsche, inventor of the Beetle and Porsche brand (Zeller, 2019). At the time of his death there were around 115 children in the fourth and fifth generations (Viehöver, 2019). Although they all claim legitimate membership to the Ferdinand Porsche dynasty, Piëch seldom honoured their rights. Early in his career when he worked as designer in the Porsche company, he was responsible for considerable financial losses. Instead of acknowledging his role in the losses, he caused intense conflict with his siblings and cousins who also worked for Porsche (Hetzner, 2017). This conflict motivated his Porsche cousins to resign (Viehöver, 2019). Throughout his career Piëch referred to them as his counter family (Viehöver, 2019). The above examples suggest that Piëch tended to view individuals that he opposed and manipulated impersonally, as members of groups he disparaged without considering their feelings.

Although data is lacking that provides insight into the reasons he devised to justify his inconsiderate behaviour, Viehöver (2019) shed some light on this issue. As previously mentioned, he suggested that Piëch held a grudge against his cousins for attending a lax Steiner school while he, in contrast, as a dyslexic and reluctant learner had to endure a strict boarding school and university. Individuals who demonstrate the characteristics of the paranoid–paraphrenic prototype use projection as a primary defence. This implies that they typically ignore their own undesirable personality characteristics, attribute them to others, and remain hypercritical of the weaknesses of others. The data confirms Piëch’s rigid intolerance of mistakes. He usually dismissed employees who made the same mistake twice (Kacher, 2019). However, despite his own extraordinary success at VW, his career included several colossal blunders (Winton, 2019). These include projects that resulted in considerable financial loss (i.e., the Porsche racing car project), the appointment of hand-picked executives that were later dismissed (e.g., Bernd Pischetsrieder and Wendeling Wiedeking), projects that resulted in reputational damage to VW (e.g., dieselgate), and the development of cars that failed to sell (e.g., VW Phaeton, Bugatti Veyron, Audi A2 hatchback). In fact, the Phaeton, Bugatti Veyron and Audi A2 formally rank as three of the 10 biggest loss-making cars in history (Rauwald and Reiter, 2019). Despite these mistakes Piëch acted like a dictatorial manager, and portrayed an image of infallibility and harsh intolerance for the mistakes of others.

#### Intrapsychic Content Domain

This domain relates to individuals’ internalised representations of significant figures and relationships from the past (cf., object relations). Although the characteristics of the assertive-sadistic prototype best fit Piëch’s intrapsychic content, the data suggests that his characteristics overlap with those of two other prototypes, namely the *non-conforming–antisocial* and *paranoid–paraphrenic* prototypes. Millon et al. (2010) indicated that the inner representations of individuals who demonstrate the characteristics of the assertive-sadistic prototype are typically characterised by aggressive and malicious attitudes. At the same time the representations lack tender emotions, internal conflicts, shame, or guilt feelings. The representations of individuals who demonstrate the characteristics of the non-conforming–antisocial prototype are typically characterised by revengeful attitudes and impulses aimed at challenging established cultural ideals and mores, as well as dishonouring personal sentiments and conventional societal attainments. The representations of individuals who demonstrate the characteristics of the paranoid–paraphrenic prototype comprise rigidly held attitudes, perceptions, and ruthless drives.

This description is supported by the biographical data. It is important to keep in mind that the collected data focuses almost without exception on Piëch’s work life. The data shed little light on his personal and family life. In fact, very little is known about his domestic life. As far as his work life is concerned, the biographical data supports the description above and suggests that Piëch generally approached people with aggression and maliciousness. He inspired fear with his domineering, dictatorial style (Ewing, 2019; Winton, 2019). There is no data indicating that he experienced or expressed tender emotions such as empathy and loyalty in some situations. Piëch admitted that he does not desire harmony (Piech, 2002). Similarly, there is no data suggesting that Piëch expressed shame or guilt feelings. Instead, the data suggests a stubborn self-assuredness in the face of accusations (Viehöver, 2019) and that Piëch typically acted as if conventional rules and regulations did not apply to him. For example, Kacher (2019) recalled an experience he had in 2002 at a prelaunch event for the VW Phaeton in Abu Dhabi. Here, Piëch urged a journalist to drive faster than 180 miles per hour despite witnessing the camera flashes of speed traps.

The characteristics of the non-conforming–antisocial prototype are typically characterised by revengeful attitudes and impulses aimed at challenging established cultural ideals, norms, and sentiments (Millon et al., 2010). Throughout most of his career Piëch followed a clear strategy to eliminate relatives and managers that he viewed as competition or as incompetent. He viewed the Porsche cousins as competition and left no stone unturned to act out his hostile attitude toward them (Viehöver, 2019). He wanted to be in charge and in the dominant position. Zeller (2019) described him as a master of intrigue who employed a range of tactics to neutralise or control opponents. Similarly, Hetzner (2017) and Schudel (2019) described him as a ruthless individual who attached no value to collegiality within the workplace.

Piëch entertained a number of rigid attitudes, drives and thoughts. Once he felt wronged in any way, he reacted with stubborn, unwavering hostility (Ewing, 2019; Horrell, 2019). In situations like that, his hostility usually would last for decades and he would maintain conflict-ridden relationships with many individuals. He was intolerant of what he perceived as incompetence Horrell, 2019). He wanted – at all costs – to get his way (Viehöver, 2019). Once he made up his mind about what he wanted to achieve, he single-mindedly pursued the objective (Piech, 2002).

#### Intrapsychic Architecture Domain

This domain relates to the organisational strength, structural cohesion and congruence, as well as functional efficacy of personality. These personality characteristics have implications for the maintenance of balance and harmony, regulation of internal conflicts, and the mediation of external pressures.

Although the characteristics of the *assertive-sadistic* prototype best fit Piëch’s intrapsychic structure, the data suggests that he also exhibited some of the characteristics of two other prototypes, namely the non-conforming–antisocial and confident–narcissistic prototypes. Millon et al. (2010) indicated that although individuals who demonstrate the characteristics of the assertive-sadistic prototype are generally able to restrain expression, powerful aggressive and sexual drives may periodically overwhelm and overrun the restraints. Individuals who demonstrate the characteristics of the non-conforming–antisocial prototype generally lack defencive operations with which to control irresponsible drives and attitudes. They exhibit a low threshold for impulsive behaviour and are intolerant of delay or frustration. Individuals who demonstrate the characteristics of the confident–narcissistic prototype, have weak coping and defencive strategies. They also tend to create egocentric inner worlds in which failures are quickly redeemed and self-pride reasserted.

This description is supported by the biographical data. The data suggests that in some contexts Piëch demonstrated considerable ability to restrain expression. He was very focussed in a career that spanned five decades and knew exactly which goals he wanted to achieve and how to go about doing that. However, the data the authors reviewed so far convincingly indicates that Piëch was much less restrained in interpersonal contexts. Here, his regular and intense outbursts achieved legendary status. Ewing (2019) contrasted Piëch’s intolerance for weakness or mistakes in others and his own apparent tolerance for questionable behaviour. Examples of these have already been provided. Viehöver (2019) stated that it seemed as if Piëch operated and lived in a world of his own, with its own rules and norms. The following example sheds some light on this characteristic. At the time of his death Piëch had 12 children with four different mothers. More specifically, Piëch had five children with his first wife (Corina von Planta), two from his relationship with the wife of his cousin (Marlene Porsche), three with the wife that survived him (Ursula Piëch), as well as two children whose mother has remained unnamed to date (Hetzner, 2017; Rauwald and Reiter, 2019). This example probably relates to the creation of an egocentric inner world that Millon et al. (2010) described.

## Conclusion

There are several factors that contribute to Piëch’s fascinating, enigmatic personality. The dark side of his personality had many facets and in the following section we summarise six that have been discussed in detail in the preceding sections. Firstly, the data indicated that Piëch was an impatient, intolerant, demanding, and ruthless individual who trusted no one at work. In fact, he seemed to have had very limited desire for close, cooperative, harmonious relationships. Secondly, he was a self-centred individual who exhibited a lack of empathy and an egocentric indifference to the welfare others. He viewed employees as expendable and dismissed them when they appeared incompetent, when they questioned or opposed him, or when they failed to meet his demanding standards. Thirdly, he lacked social and communication skills and as a result he brought considerable awkwardness to interpersonal situations. Fourthly, Piëch was notoriously unfriendly, rude, hostile, aggressive, cold-blooded, demanding, and bad tempered. His emotional outbursts achieved legendary status and he sustained conflicts with relatives or stakeholders for decades. Fifthly, Piëch viewed himself above the rules of social living. In fact, he held traditional or conventional morals and rules in contempt. As a result, he never showed signs of shame, regret, guilt, or remorse. Lastly, Piëch had an arsenal of manipulative tactics at his disposal. These included fear, intimidation, domination, exploitation, threats, and ruthlessness. He also demonstrated considerable skill in spreading rumours or using networks to achieve the outcomes he pursued. The dyslexic and reluctant learner, whose parents sent him to a very strict school in a neighbouring country where he had been forced for years to submit to harsh discipline, matured into an assertive, independent-minded, and forceful manager.

The preceding sections demonstrated clearly that several extraordinary characteristics balanced the dark side of his personality. Here, we summarise six of them that were discussed in detailbefore. Firstly, Piëch enjoyed challenges and he invested his energy into mastering difficult situations and problems. He was able to take charge, make things happen, and modify his environment even in times of crisis. Secondly, he was confidently competitive and enjoyed measuring himself against siblings, cousins, and opposition companies. In addition, he knew how to use competitiveness to improve teamwork and standards. Thirdly, Piëch was very focussed. He formulated clear goals, designed clear strategies, and knew what he wanted to achieve and how. He publicly acknowledged his belief that the achievement of a goal is far more important than the needs of those involved. Fourthly, he was risk-tolerant. He enjoyed innovation and was keen to push boundaries. In addition, he was willing to undertake huge projects without fear of failure. Fifthly, Piëch adopted a meticulous approach that combined clear vision with attention to detail. He obsessed about goals, production processes, quality, and maintained perfectionistic standards. When encountering inefficiency or incompetence, he became brutally demanding and confrontational. Sixthly, he employed a strong, forceful, and controlling style to lead and wield power. He was an exceptionally talented manager who could persuade more than 6,000 employees to work for the achievement of a common goal. Despite being a feared and very controversial manager, he was successful in evoking obedience and respect from subordinates. In fact, it seemed as if he gained considerable satisfaction from opportunities to dictate and manipulate others. The above characteristics contributed significantly to his exceptionally effective leadership in times of crisis as well as his success in a volatile and highly competitive industry.

Millon’s theory (2011) proved to be effective in presenting the seemingly contradictory personality characteristics of Piëch in a coherent and integrative narrative. The theory enabled the authors to formulate a coherent and convincing personality description in the place of what seemed initially like an enigmatic, inexplicable personality puzzle. Based on this psychobiographical analysis, it can be concluded that further studies using Millon’s evolutionary personality theory should be conducted on business leaders and entrepreneurs.

From a more practical perspective further study of successful business people and entrepreneurs can focus on the inter-weaving of the dark and light sides of personality characteristics that influenced their success. This may enable a cross evaluation of personalities from various industries and generate knowledge on how healthier personality characteristics in those in leadership positions in organisations may be enhanced and encouraged over more troublesome, and even dangerous, darker personally traits.

Generally, the development of academic disciplines relies to a large extent on the revision and refinement of theories and models. In this regard, psychobiographical, single-case studies of extraordinary individuals fulfil an important function by contributing to the support or criticism of theories within the field of psychology (Chezé, 2009). Detailed accounts of individual cases provide a means for stimulating new research and informing the development of theories. In the process of exploring complex, seemingly contradictory phenomena personality in a detailed, holistic, in-depth, real-life manner (Fouché and van Niekerk, 2010), psychobiographers identify problems in existing theories and generate new propositions.

This study demonstrated the positive consequences of a ruthless leadership style in the automotive sector. Piech’s style had a positive impact on motivation, quality, teamwork, productivity, and competitiveness. The findings of the study challenge researchers to investigate the mechanisms involved in this process and it challenge theorists to revise and refine leaderhip theories.

## Data Availability Statement

The raw data supporting the conclusions of this article will be made available by the authors, without undue reservation.

## Author Contributions

All authors contributed equally to writing this article. RN made a huge and outstanding contribution by reading up on Piêch’s life and work, analysing it and interpreting it based on Millon’s theory.

## Conflict of Interest

The authors declare that the research was conducted in the absence of any commercial or financial relationships that could be construed as a potential conflict of interest.

## Publisher’s Note

All claims expressed in this article are solely those of the authors and do not necessarily represent those of their affiliated organizations, or those of the publisher, the editors and the reviewers. Any product that may be evaluated in this article, or claim that may be made by its manufacturer, is not guaranteed or endorsed by the publisher.
